# Treatment of 5 dogs with immune-mediated thrombocytopenia using Romiplostim

**DOI:** 10.1186/s12917-016-0718-4

**Published:** 2016-06-10

**Authors:** Barbara Kohn, Gürkan Bal, Aleksandra Chirek, Sina Rehbein, Abdulgabar Salama

**Affiliations:** FB Veterinärmedizin, Klinik für Kleine Haustiere, Freie Universität Berlin, Oertzenweg 19 b, 14163 Berlin, Germany; Institut für Transfusionsmedizin, Charité – Universitätsklinikum, Augustenburger Platz 1, 13353 Berlin, Germany

**Keywords:** Immune thrombocytopenia, ITP, Dog, Romiplostim, Thrombopoietin, mpl

## Abstract

**Background:**

Immune thrombocytopenia (ITP) in dogs is analogous to that in humans. Romiplostim, a novel thrombopoietin receptor (TPO-R) agonist, is currently used for the treatment of refractory ITP in humans, but not in dogs. Here, we describe the response to romiplostim in five dogs with refractory ITP. Five dogs with severe and refractory ITP (three primary and two secondary) received romiplostim subcutaneously. Four dogs were administered 3–5 μg/kg and one dog received 10–13 μg/kg body weight once weekly.

**Results:**

Romiplostim was well-tolerated and administration was associated with an increase in platelet counts in all five dogs. Four of the five dogs entered remission and relapses were not observed over a follow-up period of 3–10 months.

**Conclusions:**

Romiplostim is effective in the treatment of ITP in dogs at least as well as in humans. This finding may help to develop and use new therapeutics for ITP in dogs and humans.

## Background

Immune thrombocytopenia (ITP) is a well-characterized autoimmune bleeding disease in humans that is accompanied by the immune-mediated destruction of platelets and impaired thrombopoiesis [1–4]. Comparable to humans, dogs develop ITP spontaneously [5] or secondary following infectious or neoplastic diseases [6–8]. Differential diagnosis of ITP in both species is based on the exclusion of known causes or underlying diseases [5, 9].

The current treatment options for ITP in humans and dogs are largely identical. Corticosteroid administration is generally accepted as the first-line treatment option in affected human patients and dogs [10, 11]. However, the effect of steroids is not predictable in a single patient [12]. Approximately two-thirds of human patients achieve a complete or partial response with corticosteroids, although a high proportion of patients relapse and require alternative therapy [13]. Similarly, steroid treatment may remain ineffective and may result in severe adverse reactions in dogs [8, 10, 14–16].

A second line therapy in dogs is not well-defined and may include platelet transfusions and high dose intravenous immunoglobulins (IVIgG) for acute management, or vincristine, azathioprine, mycophenolate mofetil, cyclophosphamide, cyclosporine, danazol, leflunomide, and ultimately splenectomy for long-term management and in cases of relapse or refractory ITP [8, 10, 16–19]. These treatment options are not performed analogously and there are no generally accepted guidelines on when they should be used [16]. The rates of relapse and mortality in dogs range between 9 % and 43 % [8, 10, 15, 18, 20–22].

During the last decade, significant new aspects regarding the pathogenesis and treatment of ITP in humans have been highlighted. Patients with ITP have been found to have increased platelet destruction due to autoantibodies and an impaired thrombopoiesis in the bone marrow [3, 23]. Consequently, two thrombopoietin receptor (TPO-R) agonists, romiplostim and eltrombopag, have been shown to be effective in the treatment of human ITP [24]. Romiplostim is a 59 kDa peptibody that binds to the extracellular domain of TPO-R on megakaryocytes and platelets, activates the receptor, and increases platelet counts [25]. In contrast, eltrombopag is a small molecule with a molecular weight of 442 Da, and is a non-peptide TPO-R agonist that selectively binds to the transmembrane domain of the TPO-R and increases platelet counts [26]. The safety and efficiency of TPO agonists in the treatment of ITP has been previously studied in well-designed controlled and randomized clinical trials. Eltrombopag is administered orally at a dosage ranging between 25 and 75 mg/d, and 1–10 μg/kg romiplostim is administered subcutaneously once weekly [22, 27–29]. Treatment with TPO agonists is usually indicated in patients with refractory ITP and in patients who do not adequately respond to standard therapy [22, 27].

Dogs with therapy refractory ITP are at a high risk of life-threatening bleeding. In such cases, there are no alternative therapeutic options, and affected dogs either die or are euthanized due to thrombocytopenia [5]. As ITP in dogs is largely analogous to ITP in humans, we questioned whether human TPO agents such as the Food and Drug Administration (FDA) approved human TPO-R agonists can be used as a new therapeutic measure in dogs with ITP that cannot be controlled by standard therapy.

## Methods

Five dogs with primary or secondary ITP were admitted to the Small Animal Clinic at the Freie Universität Berlin between 10/2014 and 6/2015 and were treated with romiplostim. Inclusion criteria were diagnosed primary or secondary ITP based on complete medical records, platelet counts < 150,000/μl and a positive platelet-bound antibody test. Primary ITP was only diagnosed, if there was no evidence of any other underlying disease or cause which might have triggered platelet destruction. In presence of an additionally positive direct Coombs’ test, Evans’ syndrome was diagnosed. Discrimination of primary and secondary forms of ITP was based on the complete diagnostic work-up that comprised of a complete blood count, blood smear evaluation, testing for erythrocyte agglutination, clinical chemistry, coagulation panel, diagnostic imaging including thoracic and abdominal radiography and ultrasonography, direct and indirect tests for infectious diseases, and immunological testing [7, 8]. The dogs had been pre-treated with prednisolone and adjunctive immunosuppressive drugs and were either non-responders or were readmitted due to a relapse. A response to therapy (complete remission) was defined as an increase of the platelet count to ≥ 150,000/μl. A relapse was defined as a decrease of the platelet count below 150,000/μl after the value had already been within reference range. A dog was classified as non-responder when, the platelet counts did not increase or did not reach values above 150.000/μl.(references) The dosage for each dog was extrapolated from human data, and was dependent on the clinical responses [30]. Consent from the owners was obtained. Romiplostim is licensed in human medicine and a comparable product is not available in veterinary medicine. Therefore this drug can be used in veterinary medicine without approval of an ethics committee. Moreover all the dogs were treated using standard therapy (best practice of veterinary care) first before using this novel agent.

## Results

Depending on the availability of romiplostim and the severity of ITP in affected dogs, treatment was initially commenced with a dosage of 3–5 μg/kg per week. Prior to treatment with romiplostim, all dogs had underwent conventional treatment (Tables 1 and 2) and had experienced one or more relapses. Treatment was either ineffective or was discontinued to avoid the development of severe side effects (e.g. in the case of ehrlichiosis). Administration of romiplostim resulted in an increase of platelet counts within 3–6 days following the commencement of treatment in 4 of the 5 treated dogs (Table 2). The remaining dog suffered from ehrlichiosis and hepatopathy, and did not respond to the administration of 5.3 μg/kg of romiplostim. Bone marrow examination revealed the presence of numerous megakaryocytes. A dose escalation was attempted after the following 2 months and the dog received first 13 μg/kg and then 10 μg/kg after one week. Simultaneously, prednisolone was re-administered due to the deterioration of hepatopathy. One week after the second injection, an increase in platelet counts was observed (Table 2). We report here a mean length of treatment of 13.8 (min. 3, max. 35 and median 11 weeks) weeks, which were varying for each individual dog. None of the treated dogs developed any side effects. Concomitant therapy with other drugs was gradually reduced and halted in three of the dogs when the platelet count was stabilized. Interestingly, none of the five dogs relapsed during observation. Moreover, the initially given dose of romiplostim could be reduced in four cases (Table 2).

**Table 1:** Signalment and history of five dogs with primary and secondary immune thrombocytopenia treated with romiplostim

Dog	Signalment	ITP – diagnoses month/year	Recurrences	Previous immunosuppressive therapy	Therapy (in addition to immunosuppressive medication)
1	Bearded Collie 7-year-old, female 22 kg	7/2012: primary ITP	1) 10/2012 2) 3/2013 3) 9/2014 4) 10/2014	pred 1 mg/kg twice daily + MMF 8 mg/kg twice daily Recurrence 1) Pred, cyclosporine 2) only pred 3) pred, MMF, then dexamethasone 4) pred, MMF	omeprazole, sucralfate (to prevent gastrointestinal ulcers) ursodeoxycholic acid (due to increase in liver enzymes)
2	Border Collie 10-year-old, female-spayed 25 kg (obese)	2014: primary AIHA 5/2015: Evans’ syndrome (primary ITP and AIHA)	after 3 weeks	short-acting methyl-pred 10 mg/kg once, pred 0.8 mg/kg twice daily	doxycycline omeprazole, sucralfate
3	Poodle 3-year-old, male-neutered 25 kg	12/2014: Evans’ syndrome (primary ITP and AIHA)	after 3 weeks	pred 1.5 mg/kg twice daily, MMF 5 mg/kg twice daily	omeprazole, sucralfate
4	Mixed-breed 3-year-old, female-spayed, 22 kg	ITP associated with monocytic ehrlichiosis	after 4 weeks	pred 1 mg/kg twice daily	doxycycline omeprazole, sucralfate
5	Mixed-breed 2.5-year-old, female 14 kg	ITP associated with monocytic ehrlichiosis	2 weeks	pred 0.4 mg/kg once daily	chloramphenicole/imidocarb amoxicillin-clavulanic acid omeprazole, sucralfate ursodeoxycholic acid S-adenosyl-methionine

*Pred* prednisolone, *MMF* mycofenolate mofetil, *ITP* immune thrombocytopenia, *AIHA* autoimmune hemolytic anemia

**Table 2:** Romiplostim therapy in five dogs with primary and secondary immune thrombocytopenia: Dosage, response and outcome

Dog	Current therapy at the commencement of romiplostim	Platelets count x10^3^/μl	Romiplostim μg/kg (initial dose)	Response		Maintenance therapy	Outcome
				Day	Platelets count x10^3^/μl		
1	pred 0.5 mg/kg twice daily MMF 5 mg/kg twice daily	19	5	673	13226	2.3 μg romi	CR > 10 mon
2	pred 0.6 mg/kg twice daily	57	3	383 5131	8222 15332	now 2 μg/kg romi 0.1 mg/kg pred every other day^a^	CR > 3 mon
3	pred 0.6 mg/kg twice daily MMF 5 mg/kg twice daily	25	5	4710	58 189 217	2.5 μg	ITP CR but euthanized due to AIHA after 2.5 mon
4	pred 0.5 mg/kg twice daily	123	4.5	3178	5213	3.4	CR > 5 mon
5	a) pred 0.4 mg/kg once daily, doxycycline b) 2 months later: pred 0.5 mg/kg, amoxicillin	a) 4 b) 1	a) 5.3 b) 13	a) No response b) 4 11 15	7 3 115		b) Lost for follow-up

*Romi* romiplostim, *pred* prednisolone, *MMF* mycofenolate mofetil, *CR* complete remission (platelet counts > 200 × 10^3^/μl), *ITP* immune thrombocytopenia, *AIHA* autoimmune hemolytic anemia, *mon* months

^a^due to autoimmune hemolysis

## Discussion

ITP is the most common cause of severe thrombocytopenia in dogs [31]. Corticosteroids are considered as the cornerstone of treatment. However, in cases where these drugs remain ineffective, contraindicated, or may cause severe side effects, other treatment options are desirable [8, 16, 32]. Furthermore, dogs are, unlike humans, unable to verbally express themselves. Therefore, the true incidence of intolerability to immunosuppressive drugs remains obscure in the treated animals.

Romiplostim is produced by covalently linking two tandem dimers to the C-terminus of endogenous TPO. Thus, exposure of cells expressing TPO-R (BaF3-mpl) to romiplostim results in rapid tyrosine phosphorylation of mpl, JAK2, and STAT5, and stimulation of megakaryopoiesis and platelet production. Pharmacodynamic studies in animals including mice, rats, rabbits, monkeys, and dogs have shown well-tolerability, and dose-dependent increases in platelet counts [24, 27, 33]. Subsequently, well-designed human studies have been conducted in patients with chronic ITP. The drug was well-tolerated in all studies and most events were mild to moderate. Furthermore, there was no evidence of an increased risk of thromboembolic complications or development of antibodies against natural TPO. In 2008, romiplostim was licensed for the treatment of ITP in humans and long-term treatment appears to be well-tolerable [34–36].

**Fig. 1:**
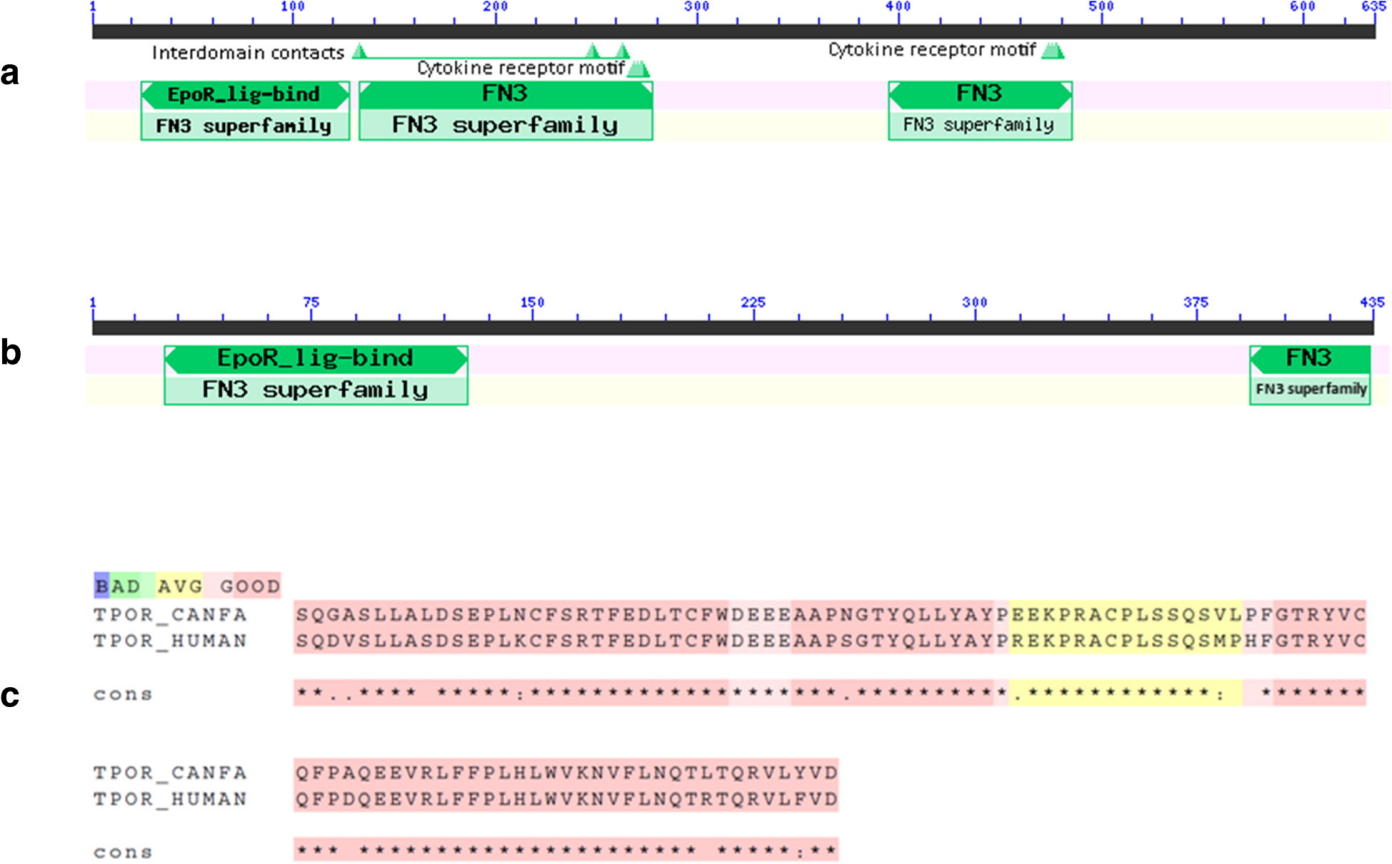
Multiple sequence analysis of thrombopoietin receptor protein sequences in canines and humans. **a** Conserved domains on the human thrombopoietin receptor gi|730980|sp|P40238.1|TPOR_HUMAN; **b** Conserved domains on the canine thrombopoietin receptor gi|73978050|ref|XP_853442.1|Canis lupus familiaris; **c** Protein sequence alignments of conserved Erythropoietin receptor, ligand binding (EpoR-lig-bind) domains in extracellular part of canines and human thrombopoietin receptor (MPL)

Depending on the phylogenetic differences of TPO-R in canines and humans, dual usage of TPO-R agonists in both species may be evolutionally encouraging or discouraging. As shown in Fig. 1, TPO-R protein sequences of canines and humans are very highly conserved at the C-terminus and the possible binding site for TPO (EpoR-lig-bind domains) is localised in this highly conserved area. As romiplostim interacts with an extracellularly located part of TPO-R and canine and human protein sequences are highly conserved, this may be the molecular basis of this therapeutic effect in canine ITP. Consistently, the safety and haematological efficiency of recombinant human TPO peptide has been demonstrated in chemotherapy-induced thrombocytopenia in dogs [37]. To date, two TPO-R agonists, romiplostim and eltrombopag, have been approved by the FDA for the treatment of ITP in humans. Although both of these drugs activate TPO-R and are used for the same indications, their binding properties and their mode of action in activating TPO-R is rather different. In contrast to romiplostim, eltrombopag interacts with the transmembrane domain of TPO-R, where the protein sequences are not phylogenetically highly conserved. Therefore, we preferred to use romiplostim as a potential candidate drug for the treatment of ITP in dogs.

In this observational study, we treated five dogs with ITP with romiplostim. All five dogs appeared not only to tolerate the drug quite well, but four of the five dogs also responded relatively quickly with a significant increase of platelet counts. One dog with secondary ITP that had not responded to prednisolone and romiplostim at a dosage of 5 μg/kg responded to a higher dosage of romiplostim. Based on the dogs’ medical history, the increase of platelet counts did not appear to be related to concomitant treatment with prednisolone.

One limitation of this pilot study is the low sample size and the inclusion of primary and secondary ITP forms. In some cases, contaminant immunosuppressive drugs was also necessary, at least, at the beginning of romiplostim therapy. Because of these limitations, dogs were treated with individual therapy protocol, inside of a clinical trial set-up. Depend on the duration of response, length of treatments were also varying for each individual dog. We report here a mean length of treatment of 13.8 weeks, whereas a mean treatment duration in human has been recently reported as 60 weeks and a maximum duration of 96 weeks [38]. Romiplostim dosage was reduced in 4 dogs (information is given in Table 2, initial dose – maintenance dose). In 3 cases romiplostim was given until the end of the observation period, one case (no. 3) was euthanized, case no. 5 was lost for follow-up.

Interestingly, the start of the increase in platelets in treated dogs appears to occur within two days after the first administration. The question whether this effect would be faster via the administration of higher doses, i.e. initially 10 μg/kg, remains to be answered in future studies. If this assumption would be true, romiplostim would be indicated as a first-line therapy in dogs requiring emergency treatment, i.e. dogs with life-threatening bleeding. The question why romiplostim appears to increase platelet counts in dogs faster than in humans remains obscure.

Romiplostim and other TPOs such as eltrombopag represent a new therapeutic measure for ITP in dogs that cannot be treated with conventional drugs or where these drugs are ineffective. Interestingly, the rapid effect is comparable with that observed in dogs treated with vincristine or IVIgG [18, 19].

Availability of romiplostim for usage in veterinary medicine may be limited by its high cost. We would therefore like to highlight one possible solution. The required amount of romiplostim for each human patient is dependent on the patient’s response and is extremely variable. Some patients may require less than 100 μg, while others may require > 500 μg. Since the drug is only available in 250 μg and 500 μg vials, it cannot be avoided that the rest of the drug will be discarded. Based on our experience, the rest of the dissolved romiplostim can be stored for several weeks (6–8 weeks) under sterile conditions at 4 ° C without a dramatic loss in biological activity. On the other hand, dogs need in total less amounts of the drug than humans. Thus, the available vials might be used in parallel for treatment of three or more dogs at the same time.

We presented here the results of a pilot study and showed that the approved human drug romiplostim may represent a novel therapeutic option in refractory dog ITP.

## Conclusions

Romiplostim is effective in the treatment of ITP in dogs at least as well as in humans. This finding may help to develop and use new therapeutics for ITP in dogs and humans.
