# Erratum to: treatment of 5 dogs with immune-mediated thrombocytopenia using romiplostim

**DOI:** 10.1186/s12917-016-0787-4

**Published:** 2016-12-30

**Authors:** Barbara Kohn, Gürkan Bal, Aleksandra Chirek, Sina Rehbein, Abdulgabar Salama

**Affiliations:** 1 Small Animal Clinic, Veterinary Medicine at the Freie Universität Berlin, Oertzenweg 19b, Berlin, 14163 Germany; 2 Institute of Transfusion Medicine, Charité – Universitätsklinikum Berlin, Augustenburger Platz 1, Berlin, 13353 Germany

## Erratum:

n.b. The errors and associated corrections described in this document concerning the original manuscript were accountable to the production department handling this manuscript, and thus are no fault of the authors of this paper. Additionally, the online manuscript has now been updated with these corrections accordingly.

- The original version of the manuscript [1] contained incorrectly formatted data in Table 2, rendering the numerical data in the sub-columns “Day” and “Platelets Count” nonsensical. The formatting of Table 2 has now been adjusted to display the appropriate numerical values in each cell more accurately.
